# Prediction of Freezing of Gait in Parkinson's Disease Using Unilateral and Bilateral Plantar-Pressure Data

**DOI:** 10.3389/fneur.2022.831063

**Published:** 2022-04-28

**Authors:** Scott Pardoel, Julie Nantel, Jonathan Kofman, Edward D. Lemaire

**Affiliations:** ^1^Department of Systems Design Engineering, University of Waterloo, Waterloo, ON, Canada; ^2^School of Human Kinetics, University of Ottawa, Ottawa, ON, Canada; ^3^Faculty of Medicine, University of Ottawa, Ottawa, ON, Canada; ^4^Centre for Rehabilitation Research and Development, Ottawa Hospital Research Institute, Ottawa, ON, Canada

**Keywords:** freezing of gait, plantar-pressure, machine learning, wearable sensors, Parkinson's disease, prediction, most affected side, least affected side

## Abstract

**Background:**

Freezing of gait (FOG) is an intermittent walking disturbance experienced by people with Parkinson's disease (PD). FOG has been linked to falling, injury, and overall reduced mobility. Wearable sensor-based devices can detect freezes already in progress and provide a cue to help the person resume walking. While this is helpful, predicting FOG episodes before onset and providing a timely cue may prevent the freeze from occurring. Wearable sensors mounted on various body parts have been used to develop FOG prediction systems. Despite the known asymmetry of PD motor symptom manifestation, the difference between the most affected side (MAS) and least affected side (LAS) is rarely considered in FOG detection and prediction studies.

**Methods:**

To examine the effect of using data from the MAS, LAS, or both limbs for FOG prediction, plantar pressure data were collected during a series of walking trials and used to extract time and frequency-based features. Three datasets were created using plantar pressure data from the MAS, LAS, and both sides together. ReliefF feature selection was performed. FOG prediction models were trained using the top 5, 10, 15, 20, 25, or 30 features for each dataset.

**Results:**

The best models were the MAS model with 15 features and the LAS and bilateral models with 5 features. The LAS model had the highest sensitivity (79.5%) and identified the highest percentage of FOG episodes (94.9%). The MAS model achieved the highest specificity (84.9%) and lowest false positive rate (1.9 false positives/walking trial). Overall, the bilateral model was best with 77.3% sensitivity and 82.9% specificity. In addition, the bilateral model identified 94.2% of FOG episodes an average of 0.8 s before FOG onset. Compared to the bilateral model, the LAS model had a higher false positive rate; however, the bilateral and LAS models were similar in all the other evaluation metrics.

**Conclusion:**

The LAS model would have similar FOG prediction performance to the bilateral model at the cost of slightly more false positives. Given the advantages of single sensor systems, the increased false positive rate may be acceptable to people with PD. Therefore, a single plantar pressure sensor placed on the LAS could be used to develop a FOG prediction system and produce performance similar to a bilateral system.

## Introduction

Parkinson's disease (PD) is a progressive neurodegenerative condition that presents various symptoms, including rigidity, bradykinesia (slowed movements), postural instability, tremor, and freezing of gait (FOG) (1). FOG is an intermittent walking disturbance, often experienced in mid-late stage PD (2) as a sudden inability to step despite the intention to walk. FOG can lead to falling, injury, and long-term effects such as fear of future falls and loss of mobility (3). Various wearable sensor-based systems have been developed (4, 5) to detect FOG using data from the freeze episode or predict freeze onset using data preceding the freeze (6–9). Cueing using auditory, visual, and tactile stimuli has been used during a freeze to help end the freeze and help the person resume walking (10–12). However, an intelligent cueing approach that generates a stimulus before the freeze, based on freeze prediction, is preferable, since it may prevent FOG from occurring.

Accelerometers and gyroscopes are the most commonly used sensors for FOG detection and prediction (4, 5). FOG prediction systems often use multiple sensors of the same type mounted on various body parts (7, 9, 13–17). Given that FOG identification systems would benefit from increased wearability and simplicity, researchers have developed FOG detection systems that use everyday devices and clothing such as smartphones (18–21) and pants (22, 23). However, noise from sensor movement relative to the body can adversely affect performance. Plantar pressure insole sensors that can be easily worn in a shoe have also been effective for FOG detection (24, 25) and prediction (26–28) and have advantages in terms of wearability and simplicity. In addition to sensor type considerations, attempts have been made to reduce prediction system complexity by using only a single sensor input, such as a single shank-mounted accelerometer (29) or a waist-mounted inertial measurement unit (IMU) (30). A single-sensor system would eliminate the need for multisensor synchronization, reduce the number of sensors worn, reduce the amount of data to acquire and process, and may be more acceptable to end users. However, additional study is required to determine if single-sensor FOG prediction systems could produce models comparable in performance to multisensor systems.

One approach to reduce the number of sensors would be to limit sensors to one side of the body. While single-side (7, 9, 13–15, 29) and bilateral (16) IMU sensors have been investigated for FOG prediction, the unilateral use of plantar pressure sensors compared to bilateral use has not been studied.

An important factor in using sensors on only one side of the body is that PD motor symptoms manifest asymmetrically and commonly affect one side of the body more severely (31). The most affected side (MAS) and the least affected side (LAS) are person specific and do not correspond to the dominant leg or hand. Although FOG detection systems have been effective using only waist and left leg sensor locations [e.g., using the Daphnet dataset (32)] without consideration of the MAS and the LAS, FOG prediction models have lower sensitivity and specificity than FOG detection models that used similar methods (4, 17, 25) and could be improved. While previous studies have not considered PD motor symptom asymmetry in FOG prediction models, there is a potential advantage of considering the MAS and the LAS in FOG prediction model development, especially if a single sensor is used exclusively.

Given the asymmetry in PD gait, benefits of single-sensor FOG prediction systems and ease of wearing plantar pressure insoles, there is a need to determine if single-limb insole instrumentation can be as effective as bilateral instrumentation in FOG prediction and if there is a preferred leg for plantar pressure insole instrumentation in FOG prediction. This study aimed to determine whether MAS, LAS, or bilateral plantar pressure data were most useful for FOG prediction. Identification of the most appropriate implementation approach is important in developing optimal systems for end users and guiding clinicians in setting up future FOG cueing systems.

## Materials and Methods

### Data Collection

The dataset used in this study is the same as in Pardoel et al. (25), with the data collection methods summarized here. Walking data were collected from 11 males with PD who experienced freezing at least once per week. Inclusion criteria were: ability to walk independently (without a walking aid), not have undergone deep brain stimulation, and not have conditions other than PD that impair their ability to walk. Data were collected during a single visit to the Human Movement Performance Laboratory, University of Ottawa. Ethics approval was obtained from the University of Ottawa (H-05-19-3547) and University of Waterloo (40954) and all the participants provided informed written consent. Participants were tested while on their regular antiparkinsonian medication dosage and schedule. Data collection was typically scheduled in the hours prior to the participant's next dose, so that the medication would be wearing off during testing and FOG would more likely occur. Participants provided disease history and were assessed using the New Freezing of Gait Questionnaire (NFOG-Q) and the Unified Parkinson's Disease Rating Scale (UPDRS)-Part III (motor examination). Participants were also asked whether their PD symptoms predominantly affected the right or left side of their body. Laterality and severity of symptoms were confirmed by the researcher (JN) conducting the UPDRS III.

Pressure-sensing insoles (FScan, Tekscan, Boston, Massachusetts, USA) were used for plantar pressure measurement during walking and data were recorded at 100 Hz. FScan insoles are thin (<1 mm) and flexible, with a resolution of 3.9 pressure-sensing cells per cm^2^. Prior to data collection, a new pair of insoles were equilibrated using a pressurized air bladder and trimmed to fit inside the participant's regular shoes. Immediately before starting the walking trials, the sensors were calibrated by having the participant stand on one foot, transfer their entire weight to the other foot, and repeat this starting with the second foot. The walking trials were video recorded using a smartphone camera (30 Hz).

**Figure 1:**
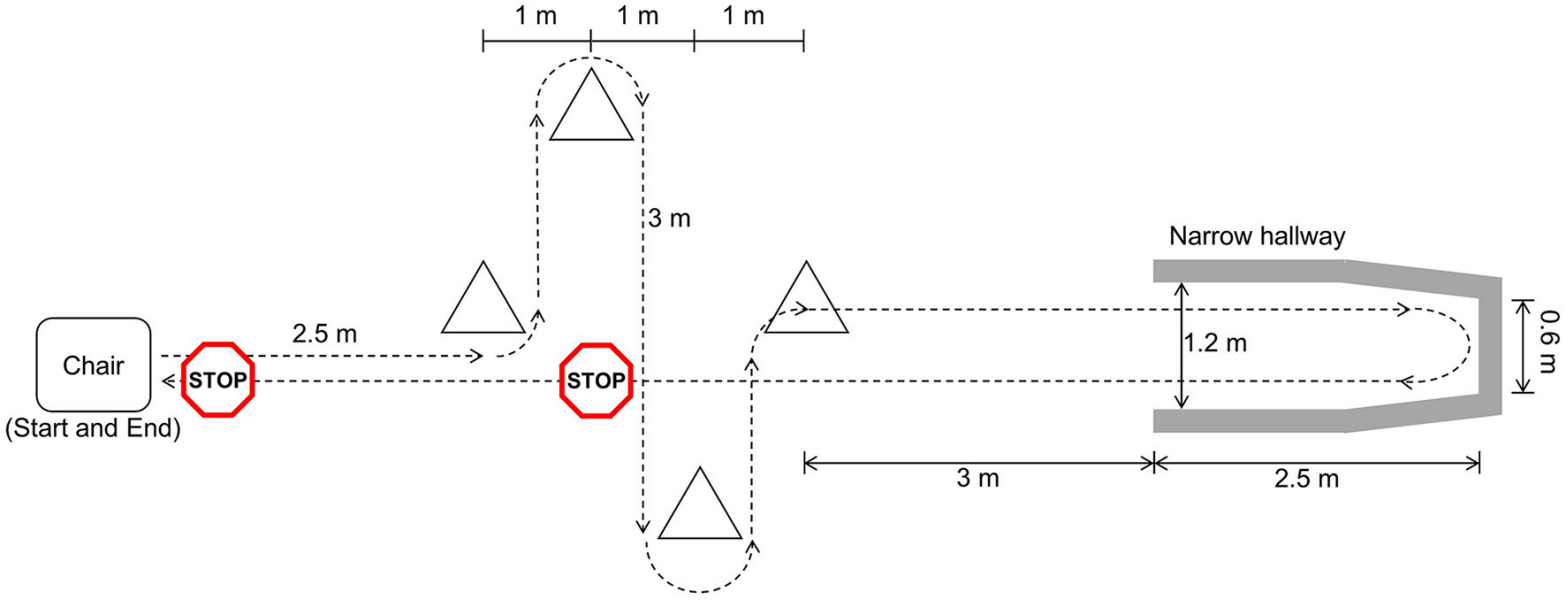
Freeze-inducing path used in walking trials [reprinted from Pardoel et al. (27), adapted from Pardoel et al. (25)].

Participants walked a freeze-inducing path up to 30 times (Figure 1). The walking path included one 90° and one 180° turn in each direction around the cones. The path also included a 180° turn in a narrow hallway. Prior to data collection, participants were asked which turning direction is most likely to cause freezing. This direction was selected for each participant as the primary turning direction in the narrow hallway. In some cases, participants were asked to change the turn direction after some trials did not produce a freeze. Additional physical and verbal tasks were performed simultaneously to increase likelihood of freezing. The physical task involved carrying a plastic tray with objects on it and the verbal task consisted of continuously saying words out loud beginning with a specific letter.

### Data Labeling

Following data collection, the video and plantar pressure data were synchronized and labeled using a custom MATLAB 2019b program. Synchronization was achieved by performing a single leg stomp at the beginning of each trial and confirmed using multiple heel strike events. During data collection, FOG episodes were identified and offline labeling was later performed by researcher SP to refine the FOG onset and termination times. In cases of uncertainly, a second labeler was consulted. Each video frame was labeled as FOG or non-FOG. The video labels were transferred to the synchronized plantar pressure data using linear interpolation to the closest timestamp.

The beginning of a freeze was defined as “the instant the stepping foot fails to leave the ground despite the clear intention to step.” The end of the freeze was defined as “the instant the stepping foot begins or resumes an effective step.” For example, a step was considered effective the instant the heel lifted from the ground, provided that it was followed by a smooth toe off with the entire foot lifting from the ground and advancing into the next step without loss of balance. As a special case, if a person froze, stopped trying to advance and remained standing, the instant that the participant stopped trying to advance was considered the end of the freeze. This was determined by the complete absence of foot movement and known FOG characteristics such as trembling of the knee, medial-lateral weight shifting, or attempt at shuffling. Furthermore, gestures and facial expressions clearly indicated that the participant was no longer trying to advance. Only a few of these special cases occurred. Pre-FOG labels were applied to all the data within the 2 s period immediately prior to the onset of a freeze episode and non-FOG labels were applied to all data that were not FOG or pre-FOG. If two FOG episodes were less than 2 s apart, the data between the two FOG episodes were labeled as pre-FOG. The 2 s pre-FOG duration represents the duration of approximately two strides and has been sufficient for FOG prediction in previous studies (8, 27). Furthermore, 2 to 3 s pre-FOG durations have led to higher pre-FOG classification accuracy than longer pre-FOG durations (17).

### Data Windowing

**Figure 2:**
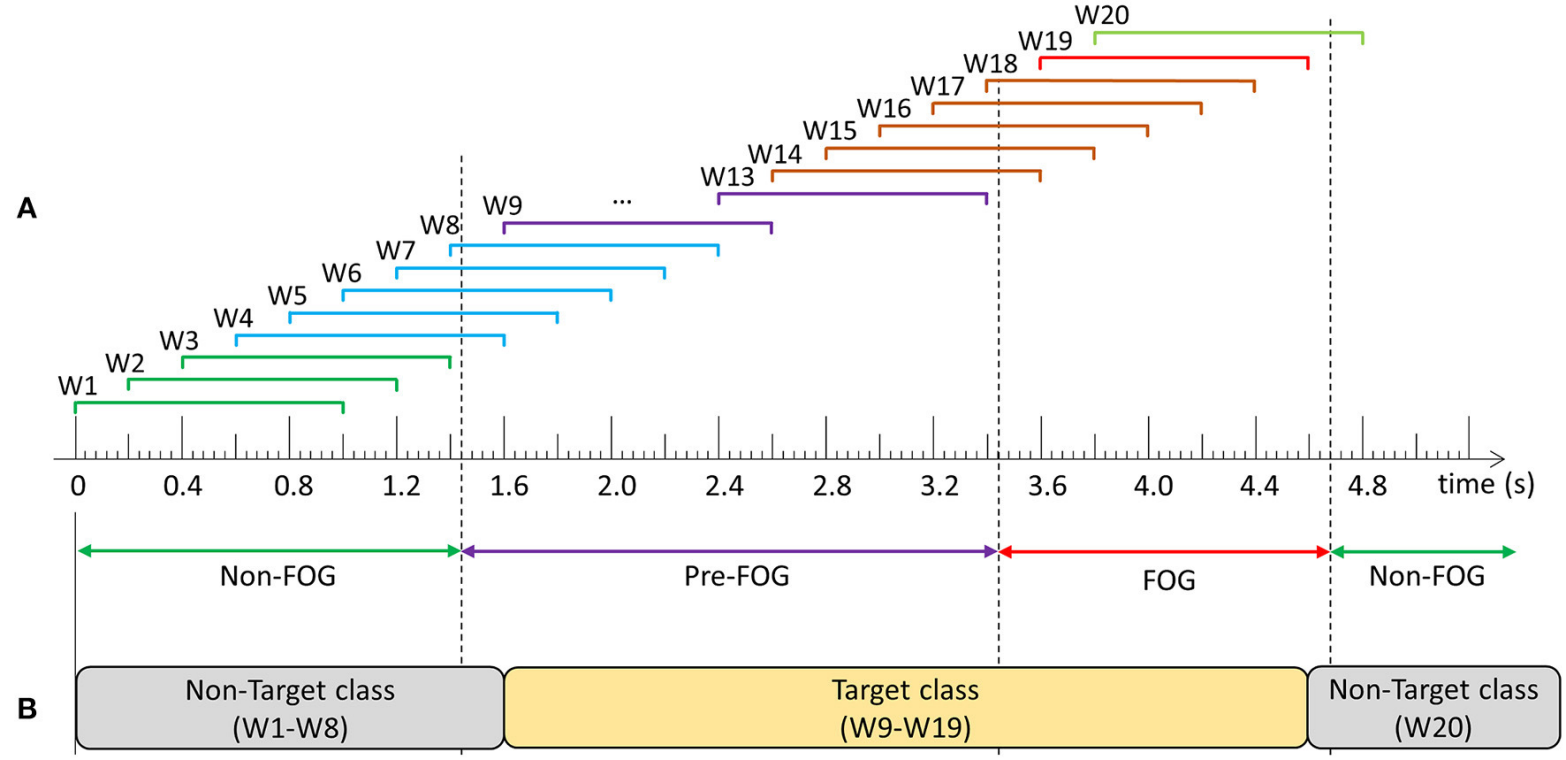
Example of data windowing and target class compositions: **(A)** Windows W1–W3 contain: non-freezing of gait (FOG) data only, W4–W8: non-FOG and pre-FOG data, W9–W13: pre-FOG data only, W14–W18: pre-FOG and FOG data, W19: FOG data only, and W20: FOG and non-FOG data, **(B)** Prediction model class composition.

Following data collection and labeling, data for each walking session were windowed using a 1 s window with a shift of 0.2 s between consecutive windows (Figure 2A). Prior to classifier model development, windows were grouped into target and non-target classes and models were trained to differentiate between the classes. The objective was to develop a single model that could predict and detect FOG. Therefore, the target class included data windows containing purely pre-FOG data (W9–W13), windows containing both the pre-FOG and FOG data (W14–W18), and purely FOG data (W19) (Figures 2A,B). The non-target class included all the other windows (W1–W8, W20).

### Feature Extraction and Ranking

Features were calculated from each data window and used to train FOG prediction models. Anterior-posterior and medial-lateral center of pressure (COP) positions and total ground reaction force (GRF) were extracted from the plantar pressure data. Prior to COP calculation, if the GRF of one foot accounted for less than 5% of the two foot total, the GRF was set to zero to remove noise or residual pressure during swing phase. COP velocity and acceleration were determined from the first and second derivatives of COP position. The GRF and COP position, velocity, and acceleration were used to calculate 13 time-domain features and 22 frequency-domain features (Table 1). The features used have been previously used for FOG identification (25).

**Table 1:** Plantar-pressure based features extracted from windowed data (25).

**Feature**	**Feature description**	**Source**	**Number of input parameters**	**Total features**
**Time domain features (*****n*** **= 13)**				
Number, duration, length of COP reversals	Number, length, duration of centre of pressure (COP) path direction reversals per window (*n* = 3)	(33)	2	6
Number, duration, length of COP deviations	Number, length, duration of medial-lateral COP deviations per window. Deviation is the first derivative of COP ML exceeding a threshold of ± 0.5 mm/window (*n* = 3)	(33)	2	6
CV of COP position, velocity, acceleration	Anterior-posterior (AP) and medial-lateral (ML) coefficients of variation (CV) of COP position, velocity, and acceleration (*n* = 6)	(33)	2	12
Number of weight shifts	Number of times the majority of total GRF (>50%) changed foot (*n* = 1)	-	1	1
	**Total computed features**	**25**		
**Fast Fourier transform (FFT) features (*****n*** **= 8)**				
Total power in FFT signal	Power in FFT signal per window as sum of squared amplitude (*n* = 1)	(34)	14	14
Dominant frequency	Frequency bin with highest amplitude per window (*n* = 1)	(35)	14	14
Max, min, mean	Maximum, minimum, and mean amplitude of FFT signal (*n* = 3)	(35)	14	42
Power in locomotion, freeze bands	Power under FFT curve in locomotion band (0.5–3 Hz) and freeze band (3–8 Hz) (*n* = 2)	(32)	14	28
Freeze index	Ratio of power in freeze band (3–8 Hz) and locomotion band (0.5–3 Hz) (*n* = 1)	(32)	14	14
**Total computed features**	**112**			
**Discrete wavelet transform features (*****n*** **= 14), Haar mother wavelet**				
Variance of coefficients	Variance of the detail and approximation coefficient vectors (*n* = 2)	(36)	14	28
Max, min, mean	Maximum, minimum, mean of detail and approximation coefficient vectors (*n* = 6)	(36)	14	84
Max, min, mean energy	Maximum, minimum, mean energy of detail and approximation coefficient vectors (*n* = 6)	(36)	14	84
**Total computed features**	**196**			

In total, 166 unilateral and 1 bilateral features (number of weight shifts) were extracted from the plantar pressure data, resulting in a total of 333 features. Relief-F ranking feature selection was used to determine the best features (Relief-F was found to be better for feature reduction than minimum-redundancy maximum-relevance ranking, tested in earlier experiments). For bilateral limb models, all 333 features were ranked. For the unilateral models, separate datasets were created with the 166 MAS and the 166 LAS features. Relief-F feature ranking was performed for the MAS and LAS datasets.

### Freezing of Gait Prediction Model Training

All prediction models developed in this paper used the same parameters and training methods. The only difference between the models was the input dataset and input features. Separate prediction models were trained using the top ranked 5, 10, 15, 20, 25, and 30 features from each of the MAS, LAS, and bilateral datasets. These values were based on previous testing that found no performance improvement when using more than 30 features. Additionally, using more than 30 features substantially increased model training time. Steps of 5 features were used to limit the total number of models trained and evaluated.

Each data window was classified using a binary classification model. Decision tree ensembles using random undersampling boosting (RUSBoosting) were trained. Each of the 100 trees had 5 splits. A leave-one-freezer-out cross-validation was performed, as in Pardoel et al. (27). Participants who did not freeze were always included in the training dataset and never held out as the test participant.

### Freezing of Gait Prediction Model Performance Evaluation

**Figure 3:**
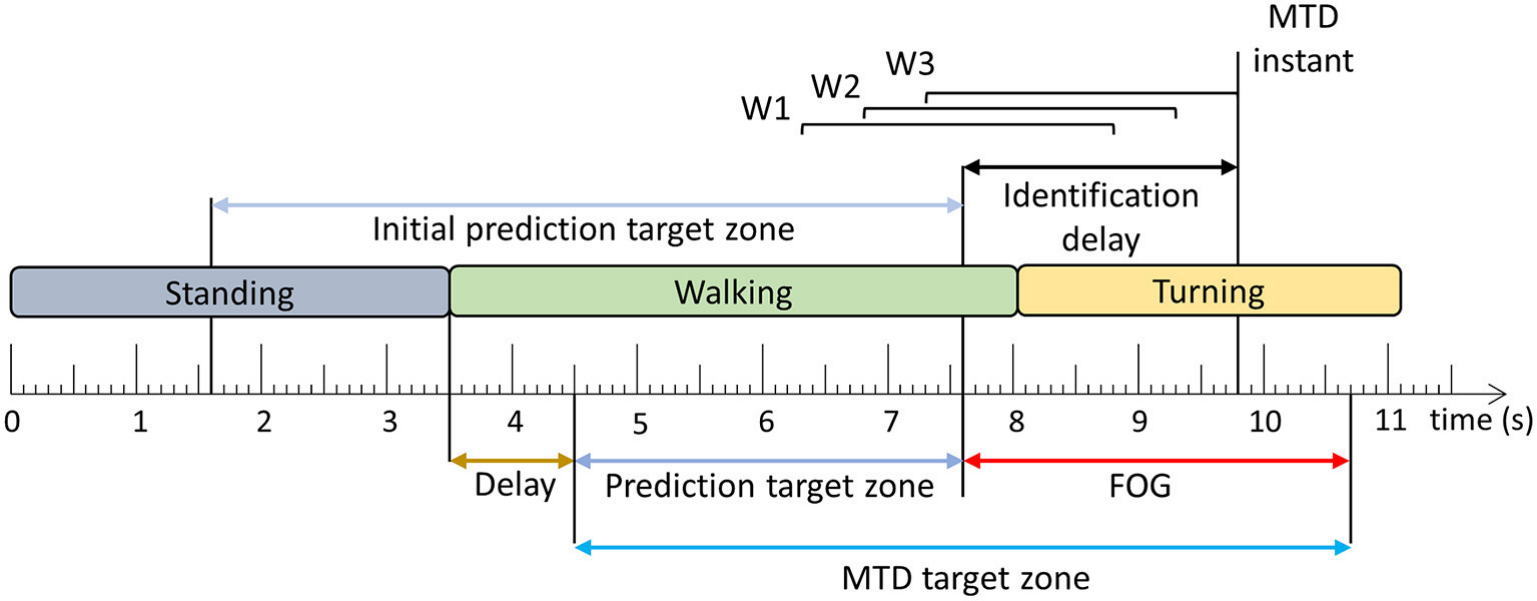
Model trigger decision example. Three consecutive windows (W1–W3) classified as the target class (Figure 2) result in a model trigger decision (MTD), where the MTD instant corresponds to the end of the third window. The FOG is successfully identified if there is a MTD instant within the MTD target zone. The time difference between FOG onset and MTD instant is the identification delay (ID). The period between the beginning of the MTD target zone and the FOG onset is the prediction target zone. The initial prediction target zone is the 6 s period before FOG onset [Adapted from Pardoel et al. (27)].

The trained models were evaluated using windows and FOG episodes similar to the evaluation in Pardoel et al. (27). The window-based evaluation compared each window classification to the ground truth label and calculated sensitivity and specificity. The FOG episode-based evaluation determined if and when each episode was detected by the model. Classification of three consecutive windows to the target class (Figure 2B) resulted in a model trigger decision (MTD) (Figure 3), which would trigger a cue if applied in a cueing system. If a MTD occurred within the MTD target zone (explained below), then the corresponding FOG episode was successfully identified. Identification delay (ID) was the time between FOG onset and a successful MTD identification. A negative ID indicated that the model predicted the FOG episode before onset and a positive ID indicated that the model detected the FOG episode after onset.

In the literature, pre-FOG gait has been identified 3 steps prior to FOG onset (37) and predictions have been reported 4–5 s in advance (7, 38) of FOG. Furthermore, model classification target zones have been defined as 8 s prior to FOG onset (9). In this paper, the MTD target zone was specific to each FOG episode (Figure 3), based on a prediction target zone that was initially set to 6 s prior to FOG onset (Figure 3). If another FOG, stand to walk transition, or turn to walk transition occurred within the 6 s period prior to FOG onset, the prediction target zone was shortened to exclude these turning, standing, or FOG data. This ensured that false positives caused by the end of the previous FOG episode, turn to walk transition, or stand to walk transition were not mistakenly interpreted as predictions of the upcoming FOG. To ensure that the turning data were not included in the MTD target zone, a 1 s delay was used, so that the prediction target zone started 1 s after the end of the turn. Similarly, for transitions from standing to walking, a 1 s delay was used to remove periods of gait initiation from the MTD target zone (Figure 3).

Each MTD was considered to be either a true positive (within the MTD target zone) or a false positive (outside the MTD target zone). The MTD false positive rate (false positives/trial) was calculated for each participant using the number of false positives and number of trials. In this analysis, false positive MTD that occurred during standing or gait initiation were ignored. Gait initiation was defined as the first second of walking after standing. As a final step in model development, a 2.5 s no-cue interval was used, wherein MTD were ignored if they occurred <2.5 s after the previous MTD (27).

## Results

Participant information is presented in Table 2. The number of FOG episodes that occurred during turning are presented in Table 3.

**Table 2:** Participant information, questionnaire results, and number of freezing of gait (FOG) episodes experienced during testing.

**Participant**	**Age (years)**	**Years since diagnosis**	**NFOG-Q**	**UPDRS III**	**Most affected side**	**Number of FOG**
P01	67	16	14	10	Right	49
P02	80	11	21	20	Left	35
P03	71	11	17	13	Left	14
P04	64	10	4	18	Left	0
P05	70	14	20	13	Right	0
P06	68	19	22	29	Left	10
P07	78	5	15	16	Right	221
P08	70	12	17	20	Right	24
P09	80	10	18	18	Left	9
P10	80	2	4	15	Left	0
P11	72	5	19	20	Right	0
Mean (SD)	72.7 (5.5)	10.5 (4.8)	15.5 (5.9)	17.5 (4.8)		

**Table 3:** Number of FOG episodes during turns.

**Participant**	**Hallway and non-hallway**			**Hallway**		
	**MAS turn FOG**	**LAS turn FOG**	**Total (MAS and LAS) turn FOG**	**MAS turn FOG**	**LAS turn FOG**	**Total (MAS and LAS) turn FOG**
P01	22	0	22	22	0	22
P02	20	15	35	20	2	22
P03	5	7	12	5	7	12
P06	10	0	10	10	0	10
P07	31	36	67	21	0	21
P08	18	6	24	0	6	6
P09	3	4	7	0	3	3
Total	109	68	177	78	18	96

*MAS turn FOG: freeze during turning with MAS as outside (stepping) limb and LAS pivot. LAS turn FOG: freeze during turning with LAS as outside (stepping) limb and MAS pivot*.

**Figure 4:**
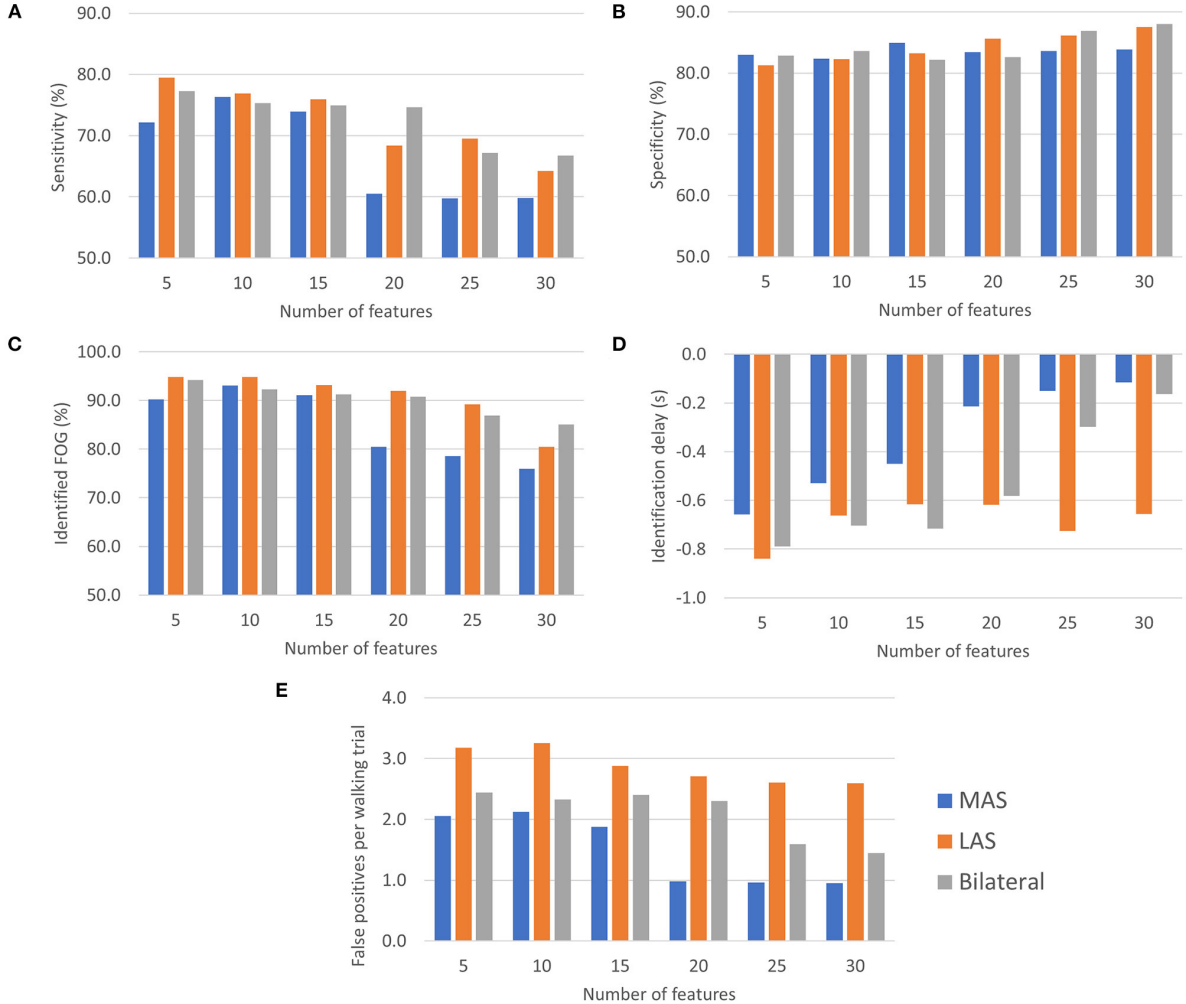
FOG prediction model performance (all values are means calculated over all held out test participants): **(A)** Sensitivity, **(B)** Specificity, **(C)** Episodes identified as a percentage of the total number of FOG episodes for each participant, **(D)** ID, and **(E)** Number of false positives per walking trial.

Freezing of gait prediction model performance for each number of features used is shown in Figure 4. All values are means calculated over all held out test participants (i.e., freezers). Overall, the highest sensitivity (79.5%) was for the LAS model with 5 features. The LAS model had the highest sensitivity for 5, 10, 15, and 25 features. The bilateral model had the highest sensitivity for 20 (74.6%) and 30 (66.7%) features.

Specificity for all MAS, LAS, and bilateral models ranged between 81.3 and 88.0%. The highest overall specificity (88.0%) was for the bilateral model with 30 features. The LAS (87.5%) and MAS (83.9%) models using 30 features also had a high specificity.

The highest percentage of identified FOG episodes ranged from 90.2 to 94.9% for all models that used 5, 10, or 15 features. For increasing numbers of features, the percentage of identified FOG decreased for all models. Overall, the highest percentage of identified FOG (94.9%) was for the LAS models with 5 features.

For each model, some FOG episodes were predicted in advance of the freeze, while other FOG episodes were detected after onset. The ID is the average of all FOG identifications for each participant. The LAS and bilateral models produced similar identification delays using 5, 10, 15, and 20 features. Overall, the earliest identifications were for the bilateral and LAS models with 5 features, which both had a −0.8 s ID. For all the models that used 5, 10, or 15 features, the ID values were between −0.4 and −0.8 s.

The MAS models had the lowest average false positive rate per walking trial for all numbers of features and the LAS models had the highest false positive rates. Overall, the lowest false positive rate was for the MAS model using 20, 25, or 30 features (1.0 false positives/trial). The highest false positive rate was for the LAS model using 10 features (3.3 false positives/trial).

Generally, using more features tended to increase specificity, decrease sensitivity, decrease percentage of identified FOG episodes, and decrease number of false positives per trial. Increasing the number of features resulted in later predictions for the bilateral and MAS models.

To select the best feature set, the model for each feature set (Figure 4) was ranked for each evaluation metric and the model (feature set) with the smallest rank sum was selected. For example, for the MAS, the model with 5 features was the third best model for sensitivity, fifth best for specificity, third best for percentage of identified FOG episodes, best (first ranked) for ID, and fifth best for false positive rate. These ranks (3, 5, 3, 1, and 5) were summed to produce a summed score of 17 for the MAS model feature set with 5 features. This ranking was done for the MAS, LAS, and bilateral models (Table 4).

**Table 4:** Rank sum for each combination of dataset and number of features.

**Dataset**	**Number of input features**					
	**5**	**10**	**15**	**20**	**25**	**30**
MAS	17	16	12^*^	19	21	20
LAS	14^*^	18	20	20	15	18
Bilateral	13^*^	14	19	20	19	20

* *Indicates the model selected as best*.

According to the ranking, the best MAS model used 15 features. The best LAS and bilateral models both used 5 features. The features used in the best models are given in Table 5. To examine model performance for each participant, the cross-validation results for the best MAS, LAS, and bilateral models are presented in Tables 6–8.

**Table 5:** Features used for the best most affected side (MAS), least affected side (LAS), and bilateral models.

**MAS** **15 features**	**LAS** **5 features**	**Bilateral** **5 features**
Number of AP COP path reversals	Number of AP COP path reversals	Dominant frequency of COP velocity AP for right leg
Dominant frequency of COP velocity AP	Power in freeze band (3-8 Hz) of COP velocity AP	Number of AP COP path reversals for left leg
Dominant frequency of COP velocity ML	Dominant frequency of COP velocity AP	Number of AP COP path reversals for right leg
Mean energy of WT aC of COP position AP	Power in freeze band (3-8 Hz) of COP position AP	Dominant frequency of COP velocity ML for right leg
Number of ML COP path deviations	Dominant frequency of COP acceleration AP	Mean energy of WT aC of COP position AP for right leg
Mean WT aC of COP position AP		
Power in freeze band (3-8 Hz) of COP velocity AP		
Mean WT aC of COP velocity AP		
Dominant frequency of COP acceleration ML		
Power in freeze band (3-8 Hz) of COP position AP		
Dominant frequency of COP acceleration AP		
Mean WT dC of GRF		
Max energy of WT aC of COP position AP		
Power in freeze band (3-8 Hz) of COP position ML		
Mean duration of AP COP path reversals		

*AP, anterior-posterior; ML, medial-lateral; WT, wavelet transform; aC, approximation coefficient; dC, detail coefficient*.

**Table 6:** Sensitivity and specificity results for the best MAS, LAS, and bilateral models.

**Participant**	**MAS 15 features**		**LAS 5 features**		**Bilateral 5 features**	
	**Sens (%)**	**Spec (%)**	**Sens (%)**	**Spec (%)**	**Sens (%)**	**Spec (%)**
P01	59.2	87.6	77.9	73.3	69.7	81.8
P02	73.7	87.9	70.0	86.6	71.7	86.7
P03	60.7	89.3	73.8	87.5	68.3	89.5
P06	84.0	88.3	95.1	88.6	93.5	89.6
P07	57.2	83.6	64.5	79.7	68.8	81.0
P08	88.8	80.7	92.6	85.2	89.1	79.1
P09	93.7	76.9	82.3	68.2	79.7	72.3
Mean	73.9	84.9	79.5	81.3	77.3	82.9
(SD)	14.1	4.3	10.5	7.3	9.7	5.8

*Sens, sensitivity; Spec, specificity*.

**Table 7:** Episode-based model performance for the best MAS, LAS, and bilateral models.

**Participant**	**MAS 15 features**			**LAS 5 features**			**Bilateral 5 features**		
	**EI (%)**	**ID (s)**	**FPR (FP/trial)**	**EI (%)**	**ID (s)**	**FPR (FP/trial)**	**EI (%)**	**ID (s)**	**FPR (FP/trial)**
P01	85.7	−0.14	1.5	87.8	−0.2	1.3	89.8	−0.3	1.7
P02	97.1	0.01	0.8	91.4	−0.6	1.0	94.3	−0.8	1.0
P03	78.6	−0.53	2.1	85.7	−0.6	2.6	85.7	−0.5	2.3
P06	100.0	−0.07	2.2	100.0	−0.8	1.9	100.0	−0.6	1.6
P07	76.5	0.02	1.3	99.1	−0.7	5.8	89.6	−0.4	2.7
P08	100.0	−0.85	1.5	100.0	−1.3	3.5	100.0	−1.2	2.9
P09	100.0	−1.59	3.6	100.0	−1.8	6.0	100.0	−1.7	4.9
Mean	91.1	–0.4	1.9	94.9	–0.8	3.2	94.2	–0.8	2.4
(SD)	9.8	0.6	0.8	5.9	0.5	1.9	5.5	0.4	1.2

*EI, episodes identified as a percentage of the total number of FOG episodes for each participant; ID, identification delay; FP, false positive; FPR, false positive rate. ID is reported as a participant average*.

**Table 8:** Identified FOG during turns for the best MAS, LAS, and bilateral models.

**Participant**	**MAS 15 features**		**LAS 5 features**		**Bilateral 5 features**	
	**Identified** **MAS turn FOG (%)**	**Identified** **LAS turn FOG (%)**	**Identified** **MAS turn FOG (%)**	**Identified** **LAS turn FOG (%)**	**Identified** **MAS turn FOG (%)**	**Identified** **LAS turn FOG (%)**
P01	81.8	–	86.4	–	86.4	–
P02	95.0	100.0	90.0	93.3	95.0	93.3
P03	40.0	100.0	60.0	100.0	60.0	100.0
P06	100.0	–	100.0	–	100.0	–
P07	64.5	75.0	100.0	100.0	83.9	91.7
P08	100.0	100.0	100.0	100.0	100.0	100.0
P09	100.0	100.0	100.0	100.0	100.0	100.0
Mean	83.0	95.0	90.9	98.7	89.3	97.0
(SD)	21.4	10.0	13.7	2.7	13.5	3.7

*MAS turn FOG: freeze during turning with MAS as outside (stepping) limb and LAS pivot. LAS turn FOG: freeze during turning with LAS as outside (stepping) limb and MAS pivot*.

## Discussion

The overall best model for FOG prediction was the bilateral model, with 77.3% sensitivity, 82.9% specificity, −0.8 s ID, 94.2% of FOG episodes identified, and 2.4 false positives per walking trial. The LAS model had similar results and only 0.8 more false positives per walking trial than the bilateral model. The MAS model had inferior results to the other two models, with 3.4% lower sensitivity and 3% fewer identified FOG episodes, 0.4 s later than the bilateral model.

For the bilateral model, sensitivity and specificity were lower than models in the literature, where sensors were worn on both limbs (17). A model using gyroscope data from the shins predicted FOG with 84.1% sensitivity and 85.9% specificity (17). However, the model was developed using data from only 35 FOG episodes. For comparison, the models developed in this paper used data from 362 FOG episodes. Using many FOG episodes during model training is desirable, since it can help achieve good model generalizability and thus, better classification performance when tested on previously unseen data. Other models in the literature achieved even higher sensitivity and specificity (9, 14, 15). For example, a person-specific model (i.e., model tuned to a specific individual) using an ensemble of 9 support vector machine classifiers and data from 3 IMU sensors reported 93% sensitivity and 87% specificity (9). Using the same dataset, a 3 class (pre-FOG, FOG, and non-FOG) k-nearest neighbors classifier achieved 94.1% sensitivity and 97.1% specificity (14). However, these systems were person specific and may not be generalizable to new participants or they used multiple sensors on various parts of the body and are thus, not directly comparable to this study, which used a single sensor to create person-independent models.

The sensitivity and specificity of the LAS model were comparable to other single-sensor FOG prediction studies in the literature (6, 29, 30, 39). The best LAS model performed better for FOG prediction than a similar tree-based algorithm (AdaBoosted C4.5 decision tree) that used data from a single waist-mounted IMU (30). Compared to a FOG prediction model that used electroencephalography (EEG) signals, the LAS had lower sensitivity (79.5% compared to 85.86%) and similar specificity (81.3% compared to 80.25%) (6). However, a single plantar pressure sensor could be integrated into regular footwear and could be used in a much more user-friendly wearable system than EEG sensors.

The LAS model FOG episode identification performance was very good compared to other models in the literature. The LAS model identified 94.9% of episodes, which was similar to (9), where 94% of episodes were identified and only slightly worse than a person-specific model used in Naghavi et al. (16) that identified 97.4% of episodes. The best MAS, LAS, and bilateral models in this paper all identified more than 91.0% of the FOG episodes. Furthermore, the LAS and bilateral models identified FOG 0.8 s before the freeze initiation. Thus, if used as part of a cueing system, the LAS and bilateral models would cue most of the FOG episodes, with identifications made just under 1 s prior to FOG onset.

The LAS model had higher sensitivity, earlier FOG identifications, and identified a higher percentage of FOG episodes than the MAS model. This may be explained by an increased role of the least affected limb in balance and postural stability during walking. Differences between the MAS and LAS have been identified in various motor tasks (40) and participants with PD (with and without FOG) preferentially adjusted the positioning of their least affected limb to retain balance after slipping (41). Therefore, the LAS limb may also be preferentially used for stability during walking, similar to how amputees rely on the intact limb for stability and balance (42). Postural stability and FOG are intricately related (43) and stability in freezers can be negatively affected by dual-task walking, which is a common trigger for FOG (44). Furthermore, stability and postural control in PD can be assessed using COP (45, 46). COP-based features that indicate postural instability may also indicate upcoming FOG. Therefore, if participants are preferentially using the least affected limb for stability control when walking, the link between instability and FOG may lead to the LAS being the more informative limb for FOG prediction. The connection between postural stability, FOG, and the preferential limb for stability control during walking should be further investigated.

The best MAS model had the highest specificity, lowest false positive rate, and latest predictions compared to the best LAS and bilateral models. Therefore, the MAS predicted FOG less in advance, but resulted in fewer false positive MTD. The best MAS model had 1.9 false positives per walking trial. Using the duration of each walking trial and averaging over all walking trials and all participants, the best MAS model thus produced one false positive approximately every 38 s of walking. Similarly, one false positive was produced approximately every 28 s for the bilateral model and every 24 s for the LAS model. However, since a specially designed freeze-inducing walking path was used in this paper, fewer false positives may be experienced during daily walking.

In a clinical setting, the choice of limb to use for sensor mounting and data collection may depend on the person, their FOG history, and the intervention (cueing) approach. For someone who tends to recover independently following a freeze, minimizing false positives may be more important than early cueing. Thus, instrumenting the MAS may be preferable, to benefit from the higher specificity and fewer false positives. In contrast, for someone who frequently experiences loss of balance and potential falls when freezing, the LAS may be preferable, since FOG episodes would be identified earlier and with higher sensitivity. For this person, a late or missing cue may be more disruptive to overall walking than the increased number of false positives. The type of cue may also influence the decision to instrument the MAS or LAS limb. When using a low intensity or minimally distracting cue, false positives may be better tolerated, thus supporting the use of the LAS model. An intense or potentially bothersome cue may be best used with MAS instrumentation to reduce unnecessary cueing.

While the LAS model performance was similar to the bilateral model, the bilateral model is recommended for best FOG prediction performance, since it produced fewer false positives. On the other hand, the difference between false positive frequencies (LAS 1:24 s, bilateral 1:28 s, and MAS 1:38 s) may be imperceptible to the user. Furthermore, single sensor systems can potentially be simpler, less expensive, and more user-friendly than systems with multiple sensors. These advantages may be more important than a slight decrease in false positive rate. Therefore, systems that use plantar pressure data from the LAS may be preferable to systems that use plantar pressure data from both the feet.

Of the total 362 FOG episodes, approximately half (*n* = 177) occurred during turning. Of the 177 turning FOG, 109 occurred when the MAS was the outside limb (LAS was the pivot limb). The LAS model identified 90.9% of turning FOG when the LAS was the pivot limb and 98.7% when the LAS was the outside limb. The best MAS and bilateral models correctly identified more than 95% of turning FOG when the MAS was the pivot limb and correctly identified 83.0% (MAS model) and 89.3% (bilateral model) when the MAS was the outside limb. The performance of the models could be affected by most hallway turns having the MAS as the outside limb, the imbalance in the number of turns in each direction, and the differences in number of freezes during turns for each participant. Future study may explore FOG identification for the MAS or LAS turns.

This study involved 11 participants, 7 of which froze during testing. In total, 362 FOG episodes were recorded, with 221 FOG episodes from participant P07; further study with larger datasets is recommended. A larger dataset including participants with various FOG subtypes (e.g., shuffling, trembling in place, akinetic) would allow the exploration of connections between FOG subtypes and preferred limb for instrumentation.

## Conclusion

This study compared the performance of FOG prediction models that used plantar pressure data collected from the most affected side, least affected side, and both sides. Of the RUSBoosted ensembles of decision trees trained, the best models used 5 features for the LAS and bilateral models and 15 features for the MAS model. The LAS model had higher sensitivity and identified a higher percentage of FOG episodes more in advance of the FOG onset compared to the MAS model. The MAS model had higher specificity and fewer false positives. In a system that uses a single plantar pressure sensor, the decision to instrument the LAS or MAS may be person specific. For someone who tends to recover independently from FOG, instrumenting the MAS may be preferable, since there would be fewer false positives. However, for someone who experiences loss of balance during freezing, cueing earlier may be more important than minimizing false positives, thus instrumenting the LAS may be preferable.

The LAS and bilateral model performance was similar for all evaluation metrics, except the false positive rate. The LAS model had a slightly higher false positive rate than the bilateral model. Therefore, based on prediction performance, using plantar pressure data from both feet are recommended. However, since the difference in false positive rate between the LAS and bilateral models was small, the advantages of a single sensor system may outweigh the increase in false positive rate. In practice, using a single-limb plantar-pressure based FOG prediction system could enhance wearability and compliance, since fewer sensors would need to be worn.

## Data Availability Statement

The original contributions presented in the study are included in the article/supplementary materials. Further inquiries can be directed to the corresponding author.

## Ethics Statement

This study involving human participants was reviewed and approved by the University of Ottawa (H-05-19-3547) and University of Waterloo (40954) Research Ethics Boards. The participants provided their written informed consent to participate in this study.

## Author Contributions

SP, JN, JK, and EL: conceptualization, project planning and methodology, manuscript review, and editing. SP: data analysis and manuscript first draft. All authors contributed to the article and approved the submitted version.

## Funding

This study was funded by Microsoft Canada, Waterloo Artificial Intelligence Institute and Network for Aging Research at University of Waterloo, Natural Sciences and Engineering Research Council of Canada (NSERC), Ontario Ministry of Colleges and Universities, University of Waterloo, and Weston Family Foundation through its Weston Brain Institute.

## Conflict of Interest

The authors declare that the research was conducted in the absence of any commercial or financial relationships that could be construed as a potential conflict of interest.

## Publisher's Note

All claims expressed in this article are solely those of the authors and do not necessarily represent those of their affiliated organizations, or those of the publisher, the editors and the reviewers. Any product that may be evaluated in this article, or claim that may be made by its manufacturer, is not guaranteed or endorsed by the publisher.
